# A need for confirmatory testing of isolated HBcAb-positive results in screening programs

**DOI:** 10.1097/HC9.0000000000000554

**Published:** 2024-10-03

**Authors:** Michael X. Fu, Peter Simmonds, Heli Harvala

**Affiliations:** 1 1 Nuffield Department of Medicine, University of Oxford, Oxford, UK; 2 2 Radcliffe Department of Medicine, University of Oxford, Oxford, UK; 3 3 Microbiology Services, NHS Blood and Transplant, Colindale, UK

## INTRODUCTION

Globally, an estimated 246 million individuals are chronically infected with HBV.^1^ HBV infection is the primary etiological agent of HCC and cirrhosis, both of which are associated with substantial morbidity and mortality.^2^ Screening and vaccination are cost-effective strategies for reducing the burden of HBV infection.^3^ However, HBV infection, even when asymptomatic, carries a wide range of adverse psychosocial burdens on affected individuals, impacting their mental health-related quality of life, personal relationships, education, employment, and access to health care.^2^

Chronic HBV infection is defined by the presence of HBsAg for a minimum of 6 months.^4^ The appearance of antibodies against HBsAg (anti-HBs) after a decline in HBsAg levels indicates recovery from infection, while immunocompetent individuals who have never been infected with HBV typically have positive anti-HBs titers (≥10 mIU/mL) after successful vaccination.^3^ It is estimated that at least one-third of the global population has been infected or previously exposed to HBV, demonstrated by antibodies to the HBV core antigen (anti-HBc).^4^ However, most individuals with past HBV infection remain unaware that they were ever exposed, especially those originating from an HBV-endemic country.^5^

Anti-HBc positivity in individuals without HBsAg primarily indicates recovery from HBV infection. However, isolated anti-HBc positivity (immunoglobin M) could also indicate acute HBV infection. Unfortunately, anti-HBc assay positivity, mainly in individuals testing negative for HBsAg and negative for anti-HBs, can also result from biological false reactivity (also termed false positivity or nonspecific reactivity) in assays used for anti-HBc screening, especially among individuals from low-prevalence regions who lack risk factors for HBV infection. Consequently, interpreting isolated anti-HBc reactive results can be challenging. The issue is complicated by the global increase in vaccination uptake, where false positivity should also be considered in individuals without prior HBV infection but who test positive for both anti-HBs and anti-HBc antibodies. It is imperative for guidelines to discuss optimal testing strategies for anti-HBc so that the diagnosis of a positive anti-HBc result is made with certainty, given the implications for the affected individuals and stakeholders.

## CURRENT GUIDELINES FOR ANTI-HBc TESTING

In line with the World Health Organization’s (WHO) targets for viral hepatitis elimination, the US Centers for Disease Control and Prevention (CDC) has recently recommended a one-time universal screening of all adults for HBV infection with 3 laboratory tests: HBsAg, anti-HBs, and anti-HBc.^3^ This recommendation contrasts with the American Association for the Study of Liver Diseases (AASLD) guidelines, which do not recommend general anti-HBc testing due to issues with false positivity.^6^ Indeed, a 6.2% median anti-HBc positivity rate from HBsAg-negative individuals in the United States^3^ means many individuals would be identified as anti-HBc–positive from population-wide screening.

Elsewhere, the European Association for the Study of the Liver (EASL),^7^ the Asian Pacific Association for the Study of the Liver (APASL),^4^ and the AASLD^6^ recommend testing for HBsAg and anti-HBc in all patients scheduled to receive immunosuppressive therapy or cytotoxic chemotherapy. Due to the significant morbidity and mortality associated with HBV reactivation in HBsAg-negative and anti-HBc–positive individuals, guidelines advocate that these patients undergoing high-potency immunosuppression should be initiated with antiviral prophylaxis and continued 12–18 months after cessation.

International guidelines also recommend universal anti-HBc screening for blood donors in low-endemic countries to defer potentially infectious donors who test anti-HBc–positive and prevent the transfusion of their blood components.^8^ This is because anti-HBc–positive blood donors who test HBsAg-negative with undetectable HBV DNA (termed “occult” HBV infection) have been associated with HBV transfusion transmission to immunocompromised recipients, causing significant mortality.^9^

However, these guidelines do not mention the significant issue of high false-positive rates associated with anti-HBc assays. The CDC and AASLD guidelines state that Food and Drug Administration (FDA)-approved anti-HBc assays have specificities of over 96%.^3,6^ However, specificity measures a test’s ability to identify disease-free individuals correctly. In contrast, the much lower positive predictive value (PPV) should be the metric used to delineate false-positive from true-positive results.

## ANTI-HBc FALSE REACTIVITY

The low PPV among current assays found from real-world studies should be noted in international guidelines. For example, the Abbott PRISM assay, an FDA-approved assay, was found to have a 14% false positivity rate among HBsAg-negative and initially anti-HBc reactive blood donors in Germany.^10^ A recent study found that the Architect anti-HBc II assay, another FDA-approved assay, had a PPV of only 79% when screening HBsAg-negative blood donors in England,^11^ that is, 21% of the reactive samples would be false positive. This figure contrasts with the assay’s high specificity (99.93% from 633,948 donations tested by the English blood service). We note that these studies were conducted with blood donor samples, and although blood donors may reflect the general population, the results of these studies may not be entirely generalizable. Nonetheless, even with a high specificity of 99%, no assay is perfect, and the PPV is expected to be low in low-prevalence populations. This does not mean that the assay becomes unreliable, and screening services need to interpret results in the context of the population setting where the assay is used.

When large populations with low anti-HBc seroprevalence are screened, high false positivity rates could outnumber true diagnoses. Mass screening would result in increasing numbers of individuals wrongly identified as positive. This issue becomes more urgent as community-based seroprevalence testing may be pursued more globally to achieve HBV elimination goals. Screening for anti-HBc antibodies without measures to exclude false-positive results could affect a large number of otherwise healthy individuals with no history of HBV infection. Individuals who lack an understanding of resolved HBV infection may experience anxiety and distress due to the negative public perceptions surrounding HBV positivity. It seems necessary for screening programs to introduce measures to identify and reduce notification of false-positive anti-HBc results.

## A CALL FOR AN ANTI-HBc TESTING STRATEGY

An important purpose of testing strategies is to minimize the likelihood of misdiagnoses by incorporating an optimal number of tests to ensure that the positive and negative predictive values are as close to 100% as possible. Although the CDC affirms that isolated anti-HBc–positive results may be false-positive in individuals without risk factors, they suggest that repeat testing with the same anti-HBc assay is necessary to confirm results.^3^ While a very small proportion of initially reactive results may not be repeat reactive on the same assay, many false-positive results would remain unidentified if a single-assay testing strategy was utilized. In blood donor screening in England, a low-endemic country, it has been shown that adding 1 supplemental anti-HBc assay is sufficient to almost eliminate false-positive diagnoses from an initial anti-HBc assay without affecting negative predictive value.^11^ Bayesian modeling demonstrated that false-positive diagnoses would become extremely rare when using a 2-assay testing strategy compared to a single-assay strategy, even at the lowest seroprevalences and assay sensitivities.^12^ We acknowledge that there is no gold standard anti-HBc assay, and there is a likelihood that a sample that initially tests positive in one assay but tests negative in the second assay may mean that the first assay’s result is false-positive or that the second assay’s result is false-negative.

In support of a gold standard testing strategy for anti-HBc, we propose that reflex testing should be performed on initially isolated anti-HBc–positive specimens (with or without anti-HBs from vaccination) using a different supplemental anti-HBc assay of similar sensitivity. Negative results in either assay would indicate anti-HBc negativity, while positivity in both assays would confirm true anti-HBc positivity. This strategy would be similar to the 2-assay testing strategy recommended by the WHO for HBsAg screening in low-prevalence settings.^13^

Without confirming true-positive results, data gathered and used without adjustment would inaccurately assess the global epidemiology and burden of HBV infection to guide WHO elimination targets. Further, given the costs and side effects of HBV treatment, not to mention the worry associated with HBV reactivation, the use of confirmatory assays for anti-HBc seems justified as a means to avoid prophylactic antiviral treatment from being initiated in those with unconfirmed isolated anti-HBc reactive test results.

## COMMUNICATION WITH AFFECTED INDIVIDUALS

With any gold standard testing algorithm, there is a need for appropriate communication of false-positive results to minimize the psychosocial impact of notification. In 2-assay testing strategies, individuals with false-positive results should be reassured that their health is not adversely affected. Individuals could be advised that test anomalies found in healthy individuals constitute a separate entity from true-positive results found in patients. In testing strategies that utilize only 1 anti-HBc assay, clear communication should be given to individuals testing isolated anti-HBc–positive (or with anti-HBs positivity resulting from vaccination). Here, individuals could be advised that results were not confirmed, and there is uncertainty about whether the individual had past HBV infection. Regular staff training and laboratory quality assessments should be enforced to enable robust anti-HBc laboratory testing and reduce the risk of false-positive results. Optimizing messaging through counselor training and appropriately worded standard information for individuals and their health providers is crucial.

In summary, high false-positive rates observed in anti-HBc assays in low-risk and low-endemic populations lead to unnecessary notification of apparently positive results. Policymakers have a clinical and societal duty of care to ensure accurate screening test results. Implementing a 2-assay anti-HBc testing strategy as a gold standard would avoid unnecessary additional testing, surveillance, treatment, and psychological effects on individuals testing false-positive. Incorrect seroprevalence data from poor diagnostics also impacts HBV epidemiology and elimination strategies. Effective communication is vital; stakeholders involved in HBV screening must develop notification strategies that minimize stress and anxiety for individuals who test false-positive.
